# Computational model for continuous flow of autonomous vehicles at road intersections

**DOI:** 10.1371/journal.pone.0285291

**Published:** 2023-05-04

**Authors:** Danilo Jorge dos Santos Nakoneczny, Eloy Kaviski

**Affiliations:** 1 Program of Numerical Methods in Engineering, Federal University of Paraná, Curitiba, Paraná, Brazil; 2 Department of Hydraulics and Sanitation, Federal University of Paraná, Curitiba, Paraná, Brazil; University of Shanghai for Science and Technology, CHINA

## Abstract

The increasing advances in technologies used in autonomous vehicles have improved the reliability of their controls, making them more likely to be accepted by drivers and thus more common on the streets. When all vehicles become autonomous, traffic lights will need to be more efficient. In this sense, this article presents a computational model to manage the crossing of autonomous vehicles at road intersections, so that they can flow continuously along the roads without needing to stop, except in extreme cases. Based on the developed model, we implemented an algorithm and a simulator to control the behavior of autonomous vehicles with different lengths when crossing an intersection. In order to evaluate the performance of this method, we carried out 10 thousand simulations for each combination of the intersection controller’s distances of action and vehicle group size, in a total of 600 thousand simulations. Thus, a relationship was observed between the method’s efficiency and the controller’s range, where the number of collisions was zero for distances greater than or equal to 2300 *m*. Method efficiency was also related to the average speeds at which the vehicles crossed the intersection, which was close to their average initial speed.

## Introduction

Vehicles have been improved since their creation seeking to bring more comfort and safety to their occupants. For this reason, automobile and technology companies have invested in research for the development of autonomous vehicles (AVs). Currently, in addition to studies focused on improvements in mapping, environment recognition, and AV direction [1–4], there has been room for other lines of study regarding the introduction of these vehicles in traffic, in which the interaction between AVs and pedestrians will be discussed, as well as how AVs will be able to identify whether the pedestrians intend to cross the road or not. Aiming to explore and propose solutions to resolve such conflicts, researchers [5–7] have suggested the use of protocols be inserted in the programming of such vehicles. However, pedestrians will have to follow rules and some intersections might need to have their infrastructure changed, with the possible use of sensors before the crosswalk. Despite having many challenges to overcome, AVs will provide improvements in road traffic flow, as drivers take an average of 1.3 seconds to perceive and react when the traffic light signal changes from green to yellow, or red to green [8]. Drivers waste a few seconds until they realize that the traffic light has opened and a few more seconds until they accelerate the vehicle in addition to the waiting time for the traffic light to open. Therefore, with autonomous driving, the response time to start crossing an intersection can be half the time a human driver would take, resulting in a 1.5-fold increase in vehicular traffic capacity and a 2-fold reduction in delay, as the greater capacity of a road system is more related to a consistent flow and a constant speed than to high speeds [9]. It is estimated that 75% of the world’s vehicles will be autonomous by the year 2040 [10]. This will allow the development of traffic light controllers made specifically for such vehicles, which will bring significant improvements in traffic quality, such as a great reduction in the number of stops at intersections. Searching for improvements in traffic goes beyond the benefits of reducing travel time. It reflects on a better quality of life for both the people who make up the traffic and those who are not directly involved in it. Another advantage of the decrease in the number of stops is the reduction of motor energy expenditure, which will provide longer autonomy for AV batteries. Drivers will also benefit from a traffic full of autonomous vehicles, as they will become passengers, and that will bring them better mental health by removing the stress factor created by the traffic [11]. Autonomous vehicles can be classified into six automation levels, ranging from 0 to 5, where level 0 refers to driving without automation, and level 5 refers to the most advanced system, in which the performance is equivalent to that of a driver navigating in all scenarios without any restriction [12], that is, it does not require human intervention to travel from a point of origin to its destination. This final level of automation is expected to be reached in the year 2035 [13]. Thinking about this future scenario, in this study we used the concept of space-time reservations to manage the crossing of AVs at intersections, without the need for making AV reservation requests or waiting for confirmations, as the proposed model is responsible for seeking a space-time window for each AV to cross without having to stop at the stop line. The algorithm developed to work on the Intersection Controller (IC) sends instructions to the AVs on what their speed should be at each instant, and, to ensure a successful crossing, the IC monitors the position and speed of the AVs at all times. Moreover, the model is based on a simplified intersection without considering left and right turns, such as done by [14] for a traffic control model for connected vehicles. Nonetheless, this research considers vehicles with different sizes in each simulation, which has been taken into account in another research [15], but not employed in simulations.

## Model and method

The computational model developed in this study takes into account a few situations for vehicles to cross the intersection in an intermittent and safe way, where some situations cannot occur, such as the collision of a vehicle against the rear of another, the collision of vehicles at the intersection, a vehicle exceeding the maximum permitted speed of a certain lane and a vehicle that stops unnecessarily in the lane. Therefore, to solve these situations, we performed analyzes and calculations, which are described below.

### Calculation of the distance between consecutive vehicles

**Fig 1:**
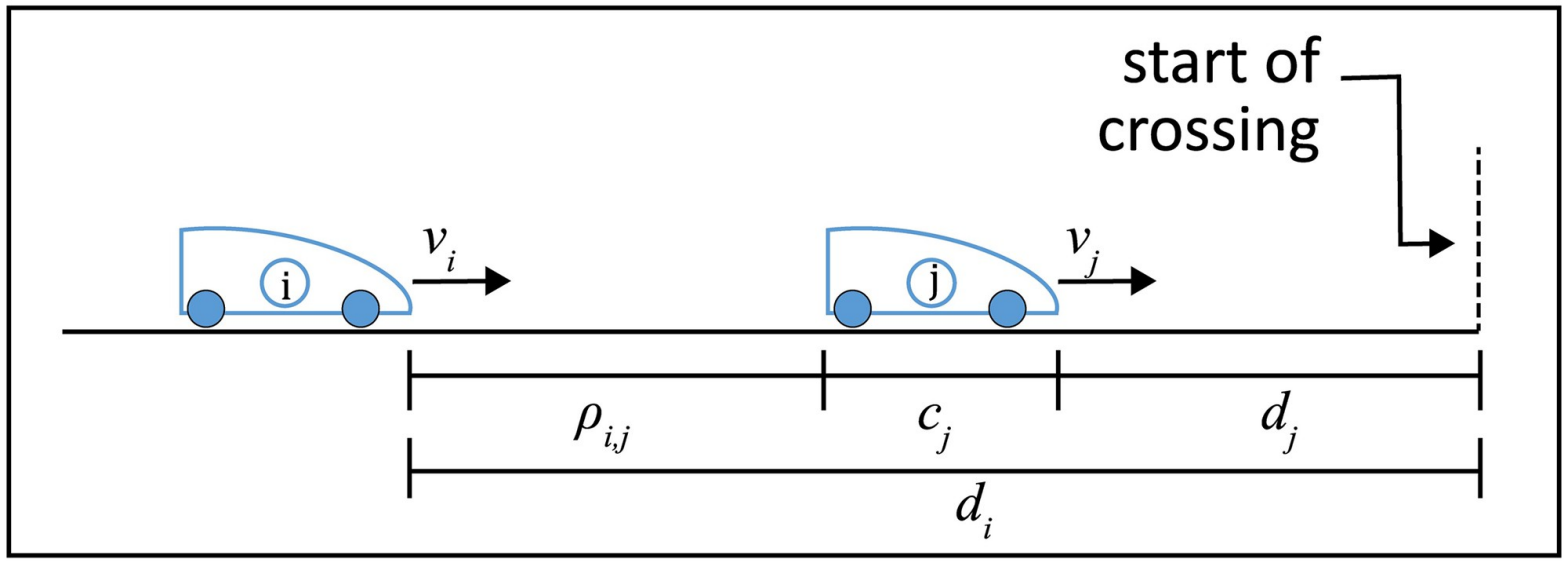
Distance between consecutive vehicles.

This calculation consists of determining the distance between two consecutive vehicles, regardless of the type of movement they perform, be it a uniformly varied movement (UVM) or a uniform movement (UM). For a better understanding of the calculation to be displayed, see Fig 1 below.

In Fig 1, *v*_*i*_ and *v*_*j*_ are the speeds of the vehicles at distances *d*_*i*_ and *d*_*j*_ from the beginning of the intersection. *c*_*j*_ is the length of the vehicle ahead. Therefore, when analyzing Fig 1, the distance between vehicles *i* and *j*, denoted by *ρ*_*i*,*j*_, can be represented by Eq (1).

$$\begin{array}{c} \rho_{i,j}=d_{i}-d_{j}-c_{j}. \end{array}$$ (1)

As the distances *d*_*i*_ and *d*_*j*_ vary as a function of time, they can be calculated using the space hourly equation, as shown below:

$$\begin{array}{c} s=s_{0}+v_{0}t+\frac{at^{2}}{2}, \end{array}$$ (2)

where

*t*: time [*s*];

*s*: distance traveled in t seconds [*m*];

*s*_0_: initial distance [*m*];

*a*: acceleration rate [*m*/*s*^2^];

*v*_0_: initial speed [*m*/*s*];

By using Eq (2), we can replace s with *d*_*i*_ and *d*_*j*_, *s*_0_ with *d*_0*i*_ and *d*_0*j*_, *v*_0_ with *v*_0*i*_ and *v*_0*j*_, and *a* with *a*_*i*_ and *a*_*j*_. As the distances *d*_*i*_ and *d*_*j*_ tend to decrease over time, the last two parts of the Eq (2) receive negative signs. Given the above, the following equations are described:

$$\begin{array}{c} d_{i}=d_{0i}-v_{0i}t-\frac{a_{i}t^{2}}{2}, \end{array}$$ (3)

and

$$\begin{array}{c} d_{j}=d_{0j}-v_{0j}t-\frac{a_{j}t^{2}}{2}. \end{array}$$ (4)

By replacing the Eqs (3) and (4) in Eq (1), we have.

$$\begin{array}{c} \rho_{i,j}=d_{0i}-d_{0j}-c_{j}+\left(v_{0j}-v_{0i}\right)t+\left(a_{j}-a_{i}\right)\frac{t^{2}}{2}, \end{array}$$ (5)

This Eq (5) indicates at each instant *t* what will be the approximation distance of two consecutive vehicles *i* and *j* on the same lane of a road. In the situation illustrated in Fig 1, three cases may occur according to the distance of the vehicles:

**1º case**—If vehicles *i* and *j* move at the same speed (*v*_*i*_ = *v*_*j*_) in a time interval, then the distance between them will remain constant during that interval.
**2º case**—If the speed of vehicle *j* is greater than the speed of vehicle *i* (*v*_*i*_ < *v*_*j*_), then the distance between them will increase over time.
**3º case**—If the speed of vehicle *i* is greater than the speed of vehicle *j* (*v*_*i*_ > *v*_*j*_), then the distance between them will decrease over time.

Among the three cases presented, the third deserves more attention as it indicates the possibility of a collision of vehicle *i* against the rear of vehicle *j*. Thus, the approximation distance between these vehicles must be monitored at all times, and it cannot be inferior to the minimum distance necessary to decelerate vehicle *i*. This minimum distance will be discussed in the following topic.

### Calculation of the minimum distance between consecutive vehicles

The calculation of the minimum distance between two consecutive vehicles in uniformly varied movement (UVM) or uniform movement (UM) is already part of the AV’s programming, however, the IC needs to perform this type of calculation to consider such a situation in the management of the AVs. It is extremely important to determine the minimum distance necessary for a vehicle to decelerate until it reaches the same speed as the vehicle ahead before a collision between them occurs. For this calculation, the Torricelli Eq (6) is considered:

$$\begin{array}{c} v^{2}=v_{0}^{2}+2a\Delta s, \end{array}$$ (6)

where

*v*: final speed [*m*/*s*];

*v*_0_: initial speed [*m*/*s*];

*a*: acceleration rate [*m*/*s*^2^];

Δ*s*: distance traveled [*m*].

Considering vehicles *i* and *j*, as represented in Fig 1, in Eq (6) we can replace the final speed *v* by the final speed at which vehicle *i* must be (which is the same as the speed of vehicle *j*) (*v* = *v*_*j*_). The initial speed *v*_0_ is the current speed of vehicle *i* (*v*_0_ = *v*_*i*_). As this calculation concerns to the 3 case shown above, where the speed of vehicle *i* is greater than the speed of vehicle *j* (*v*_*i*_ > *v*_*j*_), the acceleration rate *a* must be replaced by a deceleration rate *de* of the vehicle *i* and the traveled distance Δ*s* will indicate the minimum distance between vehicles *i* and *j* (Δ*s* = *δ*_*i*,*j*_), therefore:

$$\begin{array}{c} \delta_{i,j}=\frac{v_{j}^{2}-v_{i}^{2}}{-2\;de}. \end{array}$$ (7)

In this way, Eq (7) indicates what the minimum distance between vehicles *i* and *j* must be.

### Intersection collision analysis

**Fig 2:**
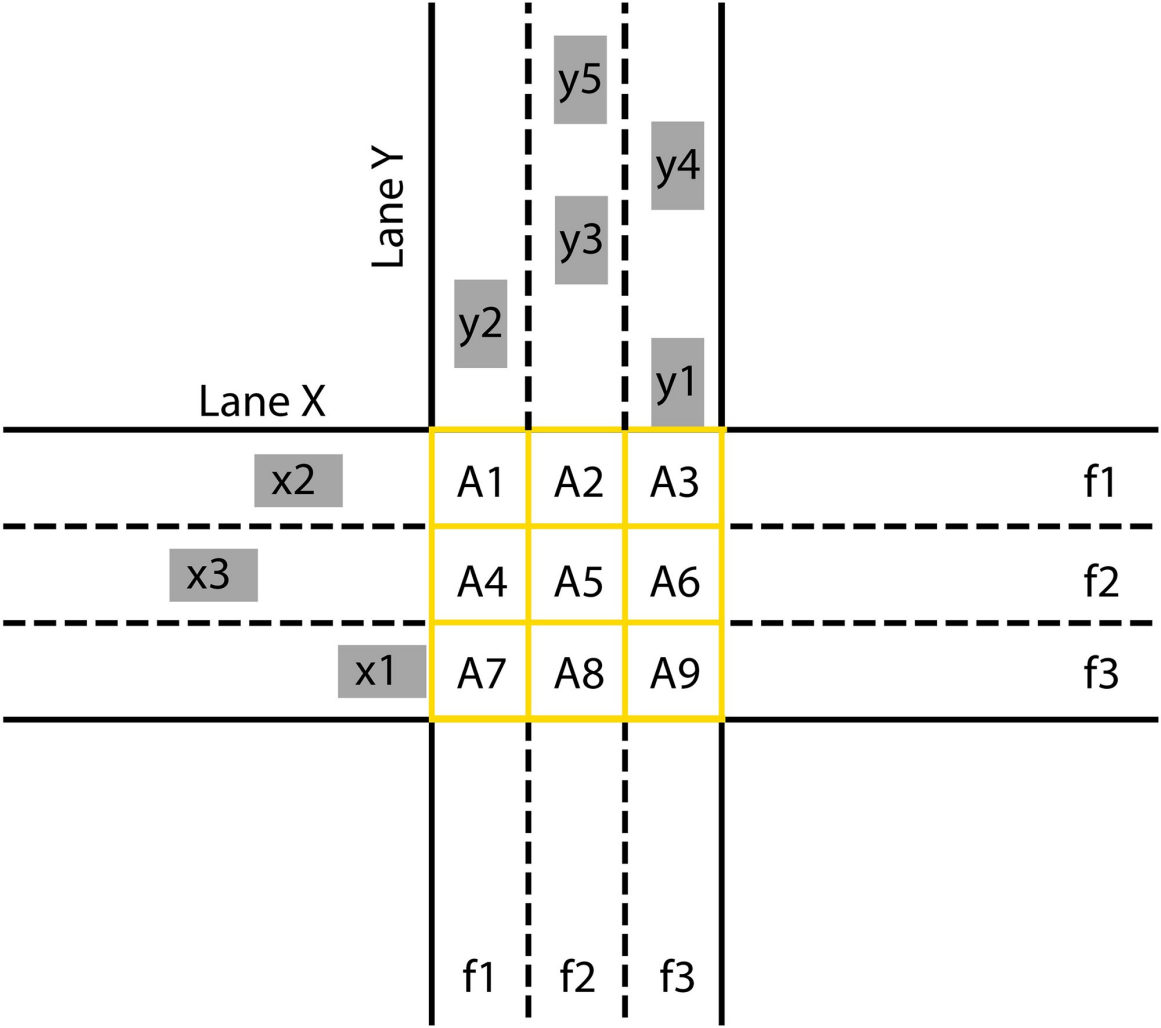
Two-way crossing with 3 lanes.

By analyzing the behavior of vehicles at an intersection where the traffic should flow intermittently, two cases can happen: one in which there will be a collision, and another in which there will not be a collision. Below, we analyze the conditions for there to be no vehicle collisions at the intersection. For this, we will consider a perpendicular intersection, where each road has three one-way lanes, as illustrated in Fig 2:

In Fig 2, x1, x2 and x3 in the grey rectangles represent the AVs on road x, while y1 to y5 represent the AVs on road y. The lanes are represented by f1, f2 and f3. The yellow-edged rectangles represented by A1 to A9 are the areas formed by the intersections of the lanes of roads x and y. We consider these areas as elements of a matrix *A*_*p*,*w*_, where *p* represents the number of the lane on road x and *w* represents the number of the lane on road y. Therefore, intending to avoid collisions at the intersection, the areas *A*_*p*,*w*_ cannot be occupied by more than one vehicle at the same time. In order to analyze whether there will be a collision or not, we can calculate the time instants at which the vehicles will enter and leave the areas that make up their respective lanes. Such time instants are represented by $tex_{A_{p,w},ix}$, $tey_{A_{p,w},iy}$, $tsx_{A_{p,w},ix}$ and $tsy_{A_{p,w},iy}$. For example, we can calculate the time instants at which vehicle x1 will enter areas A7, A8 and A9, being $tex_{A_{3,1},1}$, $tex_{A_{3,2},1}$ and $tex_{A_{3,3},1}$, respectively. The instants at which this vehicle will leave these areas are $tsx_{A_{3,1},1}$, $tsx_{A_{3,2},1}$ and $tsx_{A_{3,3},1}$. Consequently, vehicle x1 will occupy the area A7 in a time interval between $tex_{A_{3,1},1}$ and $tsx_{A_{3,1},1}$ seconds, the area A8 in a time interval between $tex_{A_{3,2},1}$ and $tsx_{A_{3,2},1}$ seconds, and finally area A9 in a time interval between $tex_{A_{3,3},1}$ and $tsx_{A_{3,3},1}$ seconds.

The instants of time are obtained by the following equations:

$$\begin{array}{c} tex_{A_{p,w},ix}=\frac{d_{ix}+\left(we-1\right)ly}{v_{ix}}, \end{array}$$ (8)

$$\begin{array}{c} tsx_{A_{p,w},ix}=\frac{d_{ix}+ws.ly+c_{ix}}{v_{ix}}, \end{array}$$ (9)

$$\begin{array}{c} tey_{A_{p,w},iy}=\frac{d_{iy}+\left(pe-1\right)lx}{v_{iy}}, \end{array}$$ (10)

$$\begin{array}{c} tsy_{A_{p,w},iy}=\frac{d_{iy}+ps.lx+c_{iy}}{v_{iy}}, \end{array}$$ (11)

where

$tex_{A_{p,w},ix}$ : instant at which vehicle *ix* on road x enters the area *A*_*p*,*w*_[*s*];

$tsx_{A_{p,w},ix}$ : instant at which vehicle *ix* on road x leaves the area *A*_*p*,*w*_[*s*];

$tey_{A_{p,w},iy}$ : instant at which vehicle *iy* on road y enters the area *A*_*p*,*w*_[*s*];

$tsy_{A_{p,w},iy}$ : instant at which vehicle *iy* on road y leaves the area *A*_*p*,*w*_[*s*];

*v*_*ix*_: current speed of vehicle *ix* on road x [*m*/*s*];

*v*_*iy*_: current speed of vehicle *iy* on road y [*m*/*s*];

*d*_*ix*_: current distance of vehicle *ix* on road x at the beginning of the intersection [*m*];

*d*_*iy*_: current distance of vehicle *iy* on road y at the beginning of the intersection [*m*];

*c*_*ix*_: length of vehicle ix [*m*/*s*];

*c*_*ix*_: length of vehicle iy [*m*/*s*];

*pe*: lane number on road x in which the vehicle will enter;

*ps*: lane number on road x from where the vehicle will leave;

*we*: lane number on road y in which the vehicle will enter;

*ws*: lane number on road y from where the vehicle will leave;

*lx*: lane width on road x [*m*];

*ly*: lane width on road y [*m*].

Note that in order for the calculation of the Eqs (8), (9), (10) and (11) to be satisfied, the speeds *v*_*ix*_ and *v*_*iy*_ cannot be equal to zero at all times, but they can be as close to it as necessary. However, for a vehicle to cross safely, one of the inequalities below must be satisfied.

$$\begin{array}{c} tsx_{A_{p,w},ix}-tey_{A_{p,w},iy}+\xi \leq 0 \end{array}$$ (12)

or

$$\begin{array}{c} tsy_{A_{p,w},iy}-tex_{A_{p,w},ix}+\xi \leq 0 \end{array}$$ (13)

When the inequality (12) is satisfied, it indicates that vehicle *ix* on road x will cross safely before vehicle *iy* on road y, as the time it takes the first to enter and leave the intersection area is at least *ξ* milliseconds shorter than the time it takes the latter to do the same. When the inequality (13) is satisfied, it indicates that vehicle *iy* will perform the crossing at least *ξ* milliseconds before vehicle *ix*.

### Computational model

**Fig 3:**
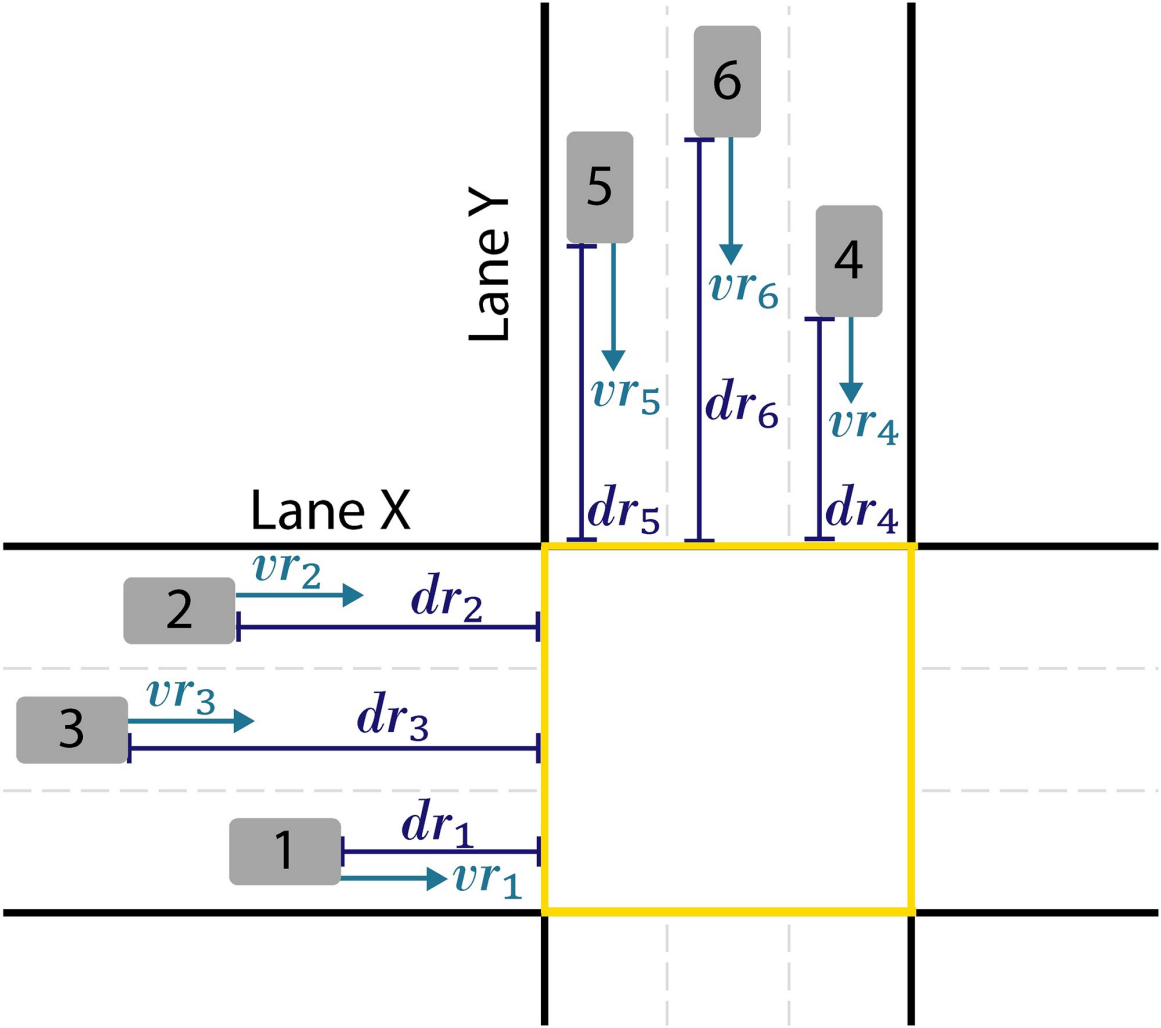
Illustration of the objective function.

The computational model is dynamic and based on a microscopic scale, as the position and speed of vehicles must be monitored individually and adjusted if necessary at each moment. The present model aims, in addition to providing a safe crossing, to minimize the travel time of vehicles within the range of the controller. Therefore, the objective function targets the maximization of the sum of the products of velocity by distance, where this distance is considered from the point at which the vehicle velocity is no longer adjusted by the controller until the end of the crossing. Fig 3 below aims to better represent this description of the objective function.

The objective function will reach its maximum value when the controller algorithm is able to determine the highest possible velocity for each vehicle, at the moment when they are at the furthest possible distance from the beginning of the intersection, so that the velocities of the vehicles remain constant until passing through the intersection completely.

$$\begin{array}{c} \text{max}\sum_{i=1}^{n}vr_{i}\;dr_{i} \end{array}$$ (14)

S.a

$$\begin{array}{c} \rho_{ix,jx}-\delta_{ix,jx}-ds\geq 0,\;ix,\;jx\in X \end{array}$$ (15)

$$\begin{array}{c} \rho_{iy,jy}-\delta_{iy,jy}-ds\geq 0,\;iy,\;jy\in Y \end{array}$$ (16)

$$\begin{array}{c} \left(tsx_{A_{p,w},ix}-tey_{A_{p,w},iy}+\xi \right)b_{i}\leq 0,\;\forall p\in P,\;\forall w\in W,ix\in X\;and\;iy\in Y \end{array}$$ (17)

$$\begin{array}{c} \left(tsy_{A_{p,w},iy}-tex_{A_{p,w},ix}+\xi \right)\left(1-b_{i}\right)\leq 0,\;\forall p\in P,\;\forall w\in W,ix\in X\;and\;iy\in Y \end{array}$$ (18)

$$\begin{array}{c} \epsilon \leq v_{ix}\leq vmax_{x},\;0<\epsilon \;\leq 10^{-3} \end{array}$$ (19)

$$\begin{array}{c} \epsilon \leq v_{iy}\leq vmax_{y},\;0<\epsilon \;\leq 10^{-3} \end{array}$$ (20)

∀*v*_*ix*_ *e* *v*_*iy*_ ∈ ℜ_+_, $\forall tex_{A_{p,w},ix}$, $tey_{A_{p,w},iy}$, $tsy_{A_{p,w},ix}$, $tsy_{A_{p,w},iy}$ ∈ ℜ_+_,*b*_*i*_ ∈ *B*.

Where, in general terms, *i* ∈ *X* ∪ *Y*, being the set *X* formed by the vehicles on road *x* and the set *Y* formed by the vehicles on road *y*. Eqs (17) and (18) cannot be satisfied simultaneously for the same pair of conflicting AVs *ix* and *iy*, so, as the variable *b*_*i*_ belongs to a binary set *B*, it is responsible for performing the controlling.

*n*: number of vehicles within the controler’s range of control, that is, *n* = ∣*X* ∪ *Y*∣;

$tex_{A_{p,w},ix}$ : instant at which vehicle *ix* on road x enters area *A*_*p*,*w*_ [*s*];

$tsx_{A_{p,w},ix}$ : instant at which vehicle *ix* on road x exits area *A*_*p*,*w*_ [*s*];

$tey_{A_{p,w},iy}$ : instant at which vehicle *iy* on road y enters area *A*_*p*,*w*_ [*s*];

$tsy_{A_{p,w},iy}$ : instant at which vehicle *iy* on road y exits area *A*_*p*,*w*_ [*s*];

*ρ*_*ix*,*jx*_: distance from vehicle *ix* to vehicle *jx* ahead on road x [*m*];

*δ*_*ix*,*jx*_: minimum distance between vehicle *ix* and vehicle *jx* ahead on road x [*m*];

*ρ*_*iy*,*jy*_: distance from vehicle *iy* to vehicle *jy* ahead on road y [*m*];

*δ*_*iy*,*jy*_: minimum distance between vehicle *iy* and vehicle *jy* ahead on road y [*m*];

*dr*_*i*_: distance vehicle *i* was from the intersection when its speed stopped being readjusted [*m*];

*ds*: safety gap between a vehicle and another vehicle ahead [*m*];

*ξ*: safety time between vehicles with conflicting movements [*s*];

*v*_*ix*_: speed of vehicle *ix* [*m*/*s*];

*v*_*iy*_: speed of vehicle *iy* [*m*/*s*];

*r*_*i*_: speed of vehicle *i* when its speed stopped being readjusted [*m*/*s*];

*v*_*max*,*x*_: maximum speed on road *x* [*m*/*s*];

*v*_*max*,*y*_: maximum speed on road *y* [*m*/*s*].

As restrictions, we have Eqs (15) and (16) assuring vehicles keep a safe distance from the others ahead, in which the distances between them cannot be shorter than the parameter ds, Eqs (17) and (18) guaranteeing there is no collision at the intersection between vehicles with conflicting movements, and finally, Eqs (19) and (20) limiting the maximum and minimum speed, in which the minimum speed is as close to zero as desired and the maximum speed cannot be higher than the one allowed on the road. The model’s decision variable stays hidden and represents the type of movement that the vehicle should perform at the next instant, which can be to accelerate, remain constant, or decelerate *a*_*de*. In this sense, the acceleration rate is the decision variable, since it will influence the vehicle’s speed at the next instant, thus impacting the instants in time in which the vehicle will enter and exit each lane of the intersection.

### Algorithm

**Fig 4:**
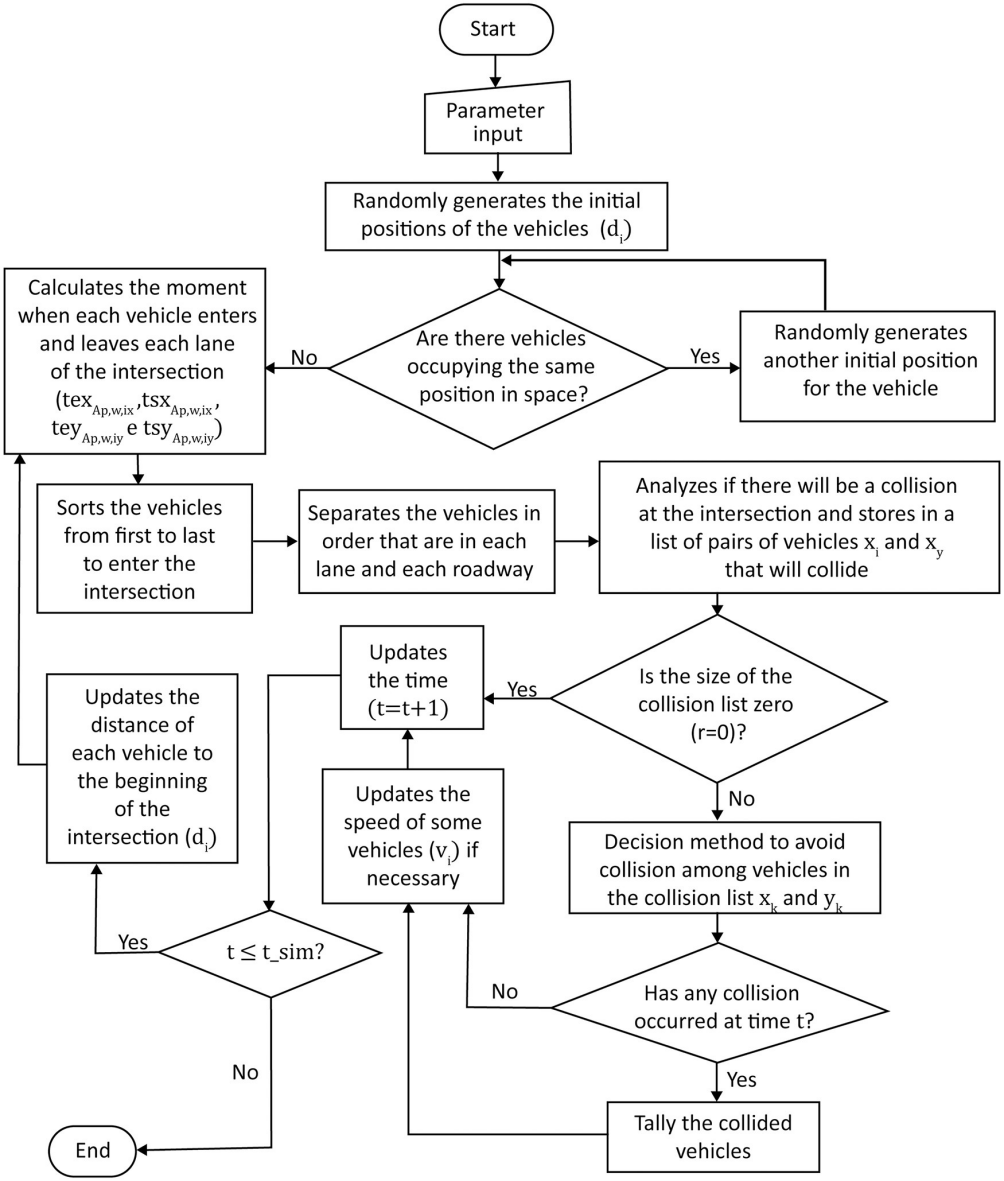
Simulation algorithm flowchart.

The solution of the computational model can be of low or high complexity and will depend on the number of AVs in each road, as well as on the speed and size of each one, and the distance that each vehicle is from the one ahead, as the model will need to be solved in real-time taking into account each one of these aspects. For this reason, the approach for this problem was to consider as a parameter a control area in which the vehicles that are within a certain distance *D* of reaching the intersection start to be monitored and managed by the intersection controller, thus becoming part of the computational model. At each second, the model is updated with the number of vehicles, the speed, and the distance that each one is from the beginning of the intersection. Since the problem is dynamic, we sought a method that solved all restrictions of the model, without the need for an optimal solution, as the search space of each iteration of the simulation is of the order of *I*^*n*^, where *I* is the number of rates of acceleration and deceleration; and *n* is the number of vehicles in the control area. For the simulation, the initial set of input data provided are the control distance *D*; the number of vehicles *n*; the number of lanes in each road *F*; width of the lanes *ly* and *lx*; the initial speed *v*_*i*_ of each vehicle; the maximum speed allowed on the road *v*_*max*,*x*_, *v*_*max*,*y*_; the vehicles’ acceleration and deceleration rates; safety gap between vehicles ds and the maximum time of each simulation *t*_*sim*_ in seconds. From this data, the software generates randomly the length *c*_*i*_ of each vehicle, the road *x* or *y*, the lane *f* in which it will be inserted, and its initial distance *d*_*i*_, which is the distance that the vehicle *i* is from the beginning of the intersection. Based on these three pieces of information we have the vehicle’s initial position. In case the position is already being occupied by another vehicle, a new position is drawn. Then, the algorithm calculates in which instants in time each vehicle will enter and exit each lane perpendicular to the one there are in $tex_{A_{p,w},ix}$, $tey_{A_{p,w},iy}$, $tsx_{A_{p,w},ix}$ and $tsy_{A_{p,w},iy}$. Such information is stored in a matrix in which each line contains the vehicle’s data, therefore, vehicles are identified by their respective line number at the matrix. The matrix’s columns store the data in the following order: *c*_*i*_, road *x* or *y*, *f*, *v*_*i*_, *d*_*i*_, $tex_{A_{p,w},ix}$, $tey_{A_{p,w},iy}$, $tsx_{A_{p,w},ix}$, $tsy_{A_{p,w},iy}$, *a*_*de* and *j*, where *a*_*de* indicates the type of movement the vehicle has to perform, if −1 it will decelerate, if 0 it will remain steady and if 1, it will accelerate, and *j* represents the identity of vehicle immediately ahead. The flowchart in Fig 4 presented below demonstrates the simulation algorithm more clearly.

**Fig 5:**
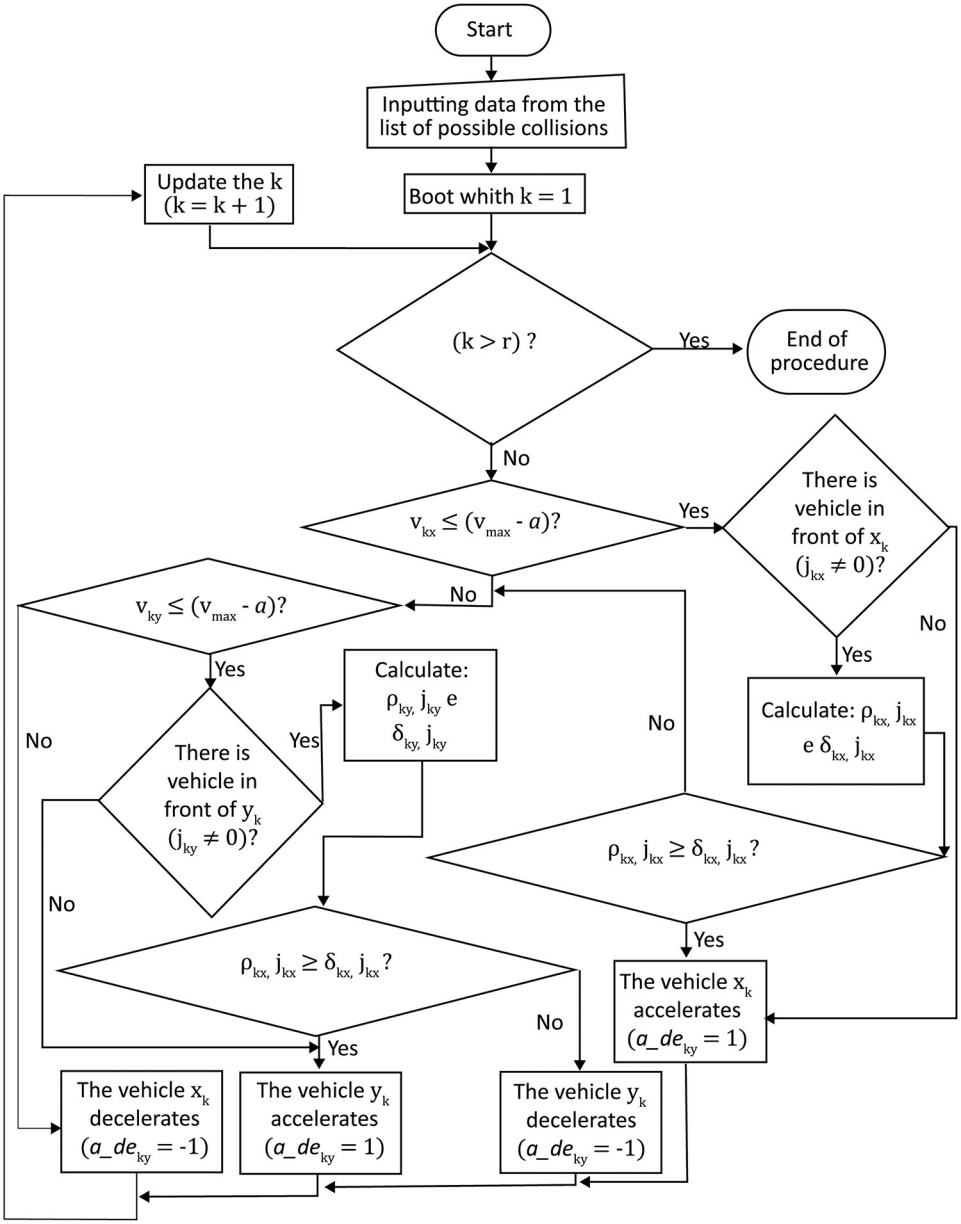
Flow chart of the decision method.

The simulation starts with *t* = 0 and a list identifying vehicles from first to last place to reach the intersection is generated. Then, vehicles are sorted by road and lane they are in, and calculations are performed to check for collisions between the vehicles that will cross the intersection. In case there is the risk of one, vehicles are identified and stored in a list of possible collisions. If the size of this list is equal to zero, then the vehicles’ speeds do not need altering and there is an increment in time, *t* = *t* + 1. If the simulation time has not ended or if not every vehicle has crossed the intersection, the distances the vehicles are from the beginning of the intersection are updated, the risk of collision is calculated again and the process repeats. In case the list of possible collisions is higher than zero, then some decisions are taken to avoid them, which basically consist in accelerating or decelerating some of the vehicles (*a*_*de* = 1 or −1). For this reason, a method that correctly makes the right decisions and chooses wisely which vehicles have to increase, decrease or keep the same speed is key for the success of the continuous traffic controller. After the method is employed, there is a check for possible collisions in the present time *t*, and if there is one, it is recorded for future analysis. For cases like this, the method needs to be further developed. The method we came up with for the decision process will be described ahead. The decision method to avoid collisions used in the problem’s simulation consists in sending a command of acceleration or deceleration (*a*_*de* = 1 or −1) for each pair of vehicles that is about to collide (*x*_*k*_ and *y*_*k*_, where *k* = 1,…,*r*), in which only one of the vehicles will have its speed modified at the instant *t*. We have that *r* corresponds to the number of collisions between each pair of vehicles, *x* and *y*, on the road, and each pair of vehicles about to collide is represented by the index *k*. The flow chart Fig 5 presented below depicts in more detail the decision process to avoid collisions.

The same vehicle can appear more than once in the list of collisions between the pairs of vehicles *x* and *y* on the road, but every time with a different vehicle. That is, there is no repetition of pairs of vehicles in the same list.

## Results

The set of data chosen for the simulations performed consists of: the distance from the control *D*, which was changed between tests; the number of lanes *F* = 3 in each road; the length of lanes *ly* and *lx*; the initial speed of each vehicle as a constant, *v*_*i*_ = 11*m*/*s*; the number of vehicles in each simulation *n* = 20, in which every vehicle is generated and distributed randomly inside a time interval equals to 200*m* for each road; the maximum speed allowed in each road, which is the same for both, *v*_*max*,*x*_ = *v*_*max*,*y*_ = 25*m*/*s*, and superior to the speed used in simulations in other works [16, 17], which used a maximum of 5m/s; the vehicles’ acceleration and deceleration rates *de*, fixed at 3*m*/*s*^2^ and 2*m*/*s*^2^ respectively, and within the range of what is expected of a human driver to achieve driving under normal circustances [18]; the safety gap between two consecutive vehicles *ds* = 1*m* and the maximum time of each simulation *t*_*sim* = 300*s*. Based on this set of data, the software generates randomly: the size *c*_*i*_ of each vehicle between 3 and 7 meters, the road and the lane f in which the vehicle will be inserted, and its initial distance *d*_*i*_, which is the distance the vehicle *i* is from the beginning of the intersection. This information gives the initial position of the vehicle, and in case the position is already occupied or the distance to another vehicle is inferior to 3 times the safety gap ds, a new position is drawn. The software’s code was developed in Pascal language using IDE Lazarus [19]. The results shown below refer to 10 thousand simulations for each control distance *D* = {100,300,500,700,…,2100,2300}*m*. We sought to analyze the efficiency of the algorithm in avoiding collisions when the control distance of the intersection controller is increased and to assess the speed averages of the vehicles when crossing the intersection in each simulation.

Table 1 presents in the first column the considered control distances *D*, in the second and third columns, the lowest and the highest speed averages of the vehicles when crossing the intersection from the 10 thousand simulations for each distance, in the fourth column, the difference between these speed averages, which is the amplitude, in the fifth column, the mean of all speed averages, and in the sixth column, the standard deviation of these speed averages.

**Table 1:** Minimum and maximum values, amplitude, mean and standard deviation of the speed averages for each distance.

*D*[*m*]	*V*_*min*_[*m*/*s*]	*V*_*max*_[*m*/*s*]	*Amp*.[*m*/*s*]	Average *V*[*m*/*s*]	St.Dev.
100	9.62	16.1	6.48	12.645	0.76353
300	6.85	15.95	9.10	11.867	0.73288
500	7.15	15.48	8.33	11.520	0.78417
700	6.66	14.05	7.39	11.202	0.85510
900	6.29	13.45	7.16	11.025	0.91381
1100	6.71	12.95	6.24	10.938	0.93476
1300	5.19	12.75	7.56	10.891	0.94390
1500	6.47	12.8	6.33	10.888	0.93606
1700	6.30	12.95	6.65	10.884	0.92080
1900	5.70	12.65	6.95	10.889	0.92072
2100	5.74	12.8	7.06	10.893	0.89714
2300	6.03	12.95	6.92	10.888	0.90899

**Fig 6:**
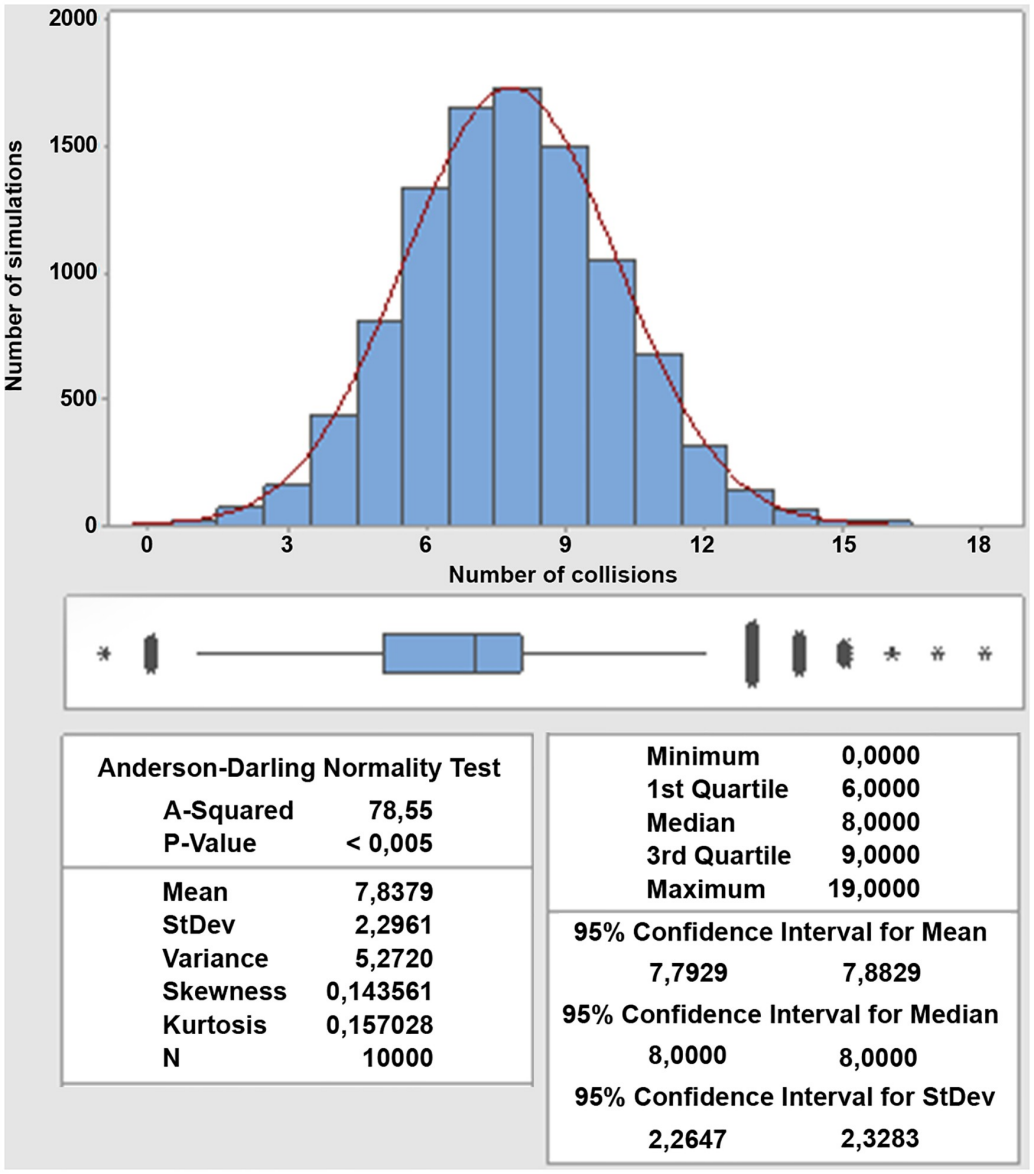
Histogram of the number of collisions without the interference of the controller.

By analyzing the values presented in the Table 1, we have that for *D* = 100, 300 *and* 500*m* the mean of the speed averages is higher than the highest values of *D*. Thus, the higher the control distance, the more the speed averages during the intersection approximate 10, 88 *m*/*s*, being close to the initial speed of 11 *m*/*s*, as can be seen in the fifth column. Looking at the minimum and maximum speed averages we can notice higher values for *D* = 100 *m* with a tendency of decrease, while the standard deviation tends to increase as *D* increases to 1500 *m*. The amplitude stays close to 7 *m*/*s* for most cases. To evaluate the algorithm’s efficiency in managing the crossing of vehicles in an intersection while avoiding collision, we forecasted the number of collisions for each simulation without the interference of the intersection controller. Fig 6 below shows this situation for a sample of 10 thousand simulations, in which the histogram tends to present a normal distribution regardless of the value of *D*.

The histogram in Fig 6 shows that approximately an average of 8 vehicles are involved in collisions per simulation, that is, 40% of the AVs simulated collide in a single intersection, with a standard deviation of 2, 296. Despite presenting a normal-distribution shape, the histogram does not follow this pattern, as the p-value is lower than 0, 05. Table 2 below shows the sum of the number of collisions without the interference of the controller, the number of effective collisions with the use of the controller, and in how many of the 10 thousand simulations there were collisions for each control distance *D* tested.

**Table 2:** Number of collisions with and without the use of the controller and number of simulations with collisions for each distance *D*.

*D* [*m*]	ini. collisions	ef. collisions	simul. with collisions
100	78269	8996	4838
300	78517	2798	1457
500	78098	864	433
700	78595	297	151
900	78570	114	69
1100	78379	83	48
1300	78261	50	25
1500	77945	47	25
1700	77867	14	12
1900	78485	8	5
2100	77939	1	1
2300	78192	0	0

**Fig 7:**
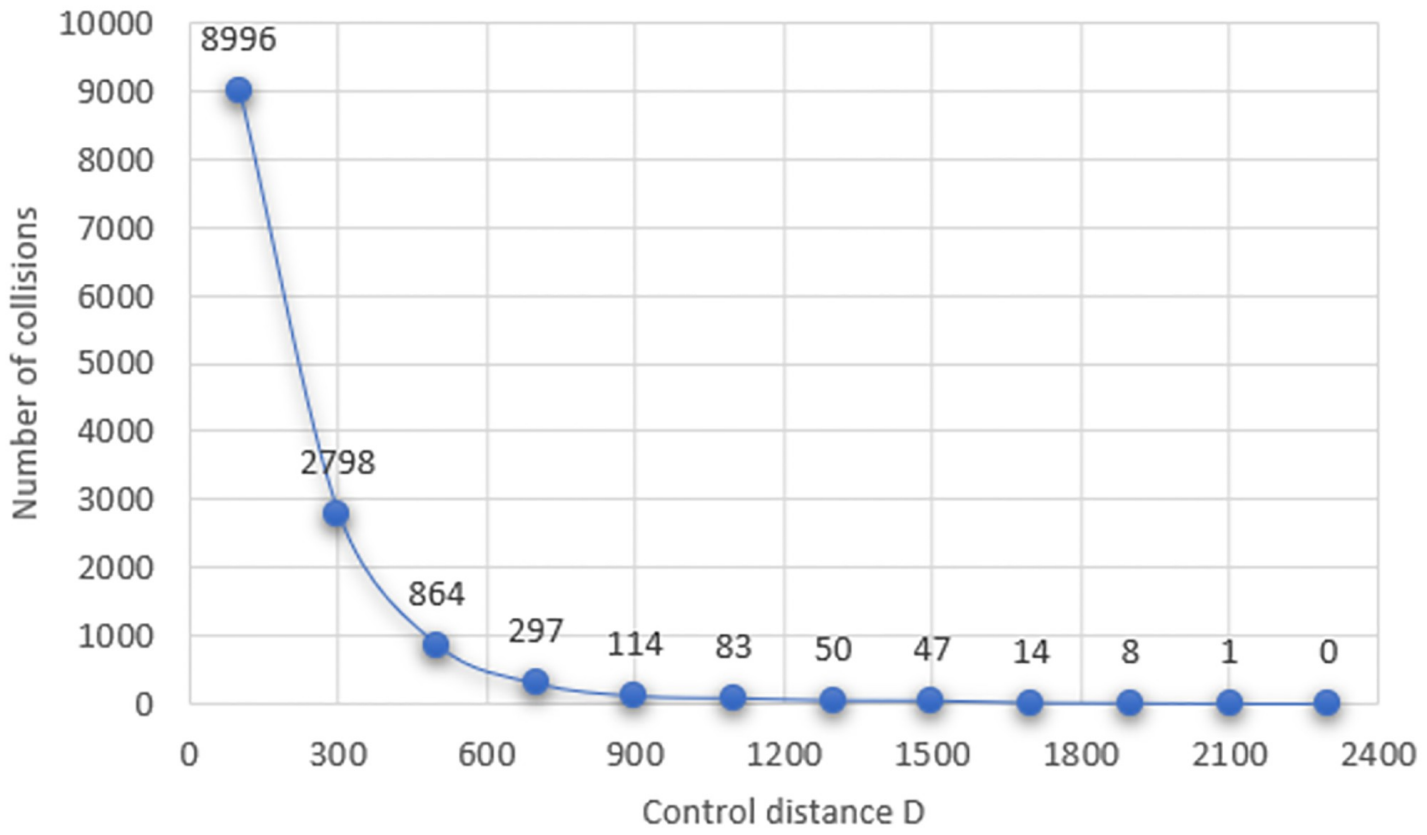
Graph of the number of collisions with the use of the controller.

In Table 2, we have that the number of collisions without the use of the controller is initially too high and does not suffer major alterations in relation to the different control distances *D*. In the last column, it is possible to notice that the number of simulations in which there were collisions decreases as the *D* increases, except for *D* equals to 1300 *m* and 1500 *m*. However, the number of vehicles that collided reduced from 50 to 47 for the same values of *D*, as shown in the third column. Next, Fig 7 presents a graph of the number of effective collisions with the use of the controller, as shown in Table 2, as a function of each value of *D* considered.

It is evident in Fig 7 the influence of the control distance on the number of effective collisions, which decreases drastically as the distance *D* increases. The time interval between the first and the last vehicle to cross the intersection was on average lower than 19 seconds for *D* = 100*m*. Thus, an average of 1, 1 vehicles cross the intersection each second, although this frequency reduces as the *D* increases.

**Fig 8:**
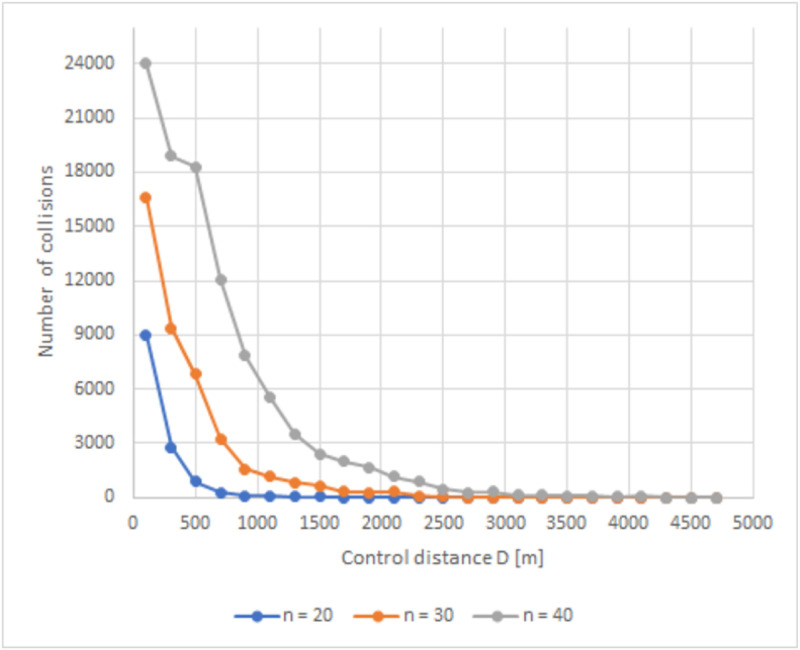
Graph of the sum of all collisions under the action the controller for each vehicle group size.

New simulations were performed, but with different demands. In addition to the simulations with 20 vehicles, we also considered *n* = 30 and 40, where the only parameters changed were the space intervals in which the vehicles are initially generated, going from 200*m* to 300*m* and 400*m* respectively, and the maximum time of each simulation, which was increased to *t*_*sim* = 2300*s* to allow all vehicles to cross the intersection, even the slowest ones. Therefore, 10 thousand simulations were performed for each vehicle group size and control distance *D* = {100,300,500,700,…,4500,4700}*m*. Fig 8 below depicts a graph of the sum of all collisions that occurred under the action of the controller in the 10 thousand simulations for each vehicle group size as a function of the control distances.

It is possible to observe in Fig 8 that the graphs of the simulations with *n* = 30 and 40 have a behavior similar to the graph for *n* = 20, as seen in Fig 7, however, with the increase in the number of vehicles, the number of collisions the controller could not avoid also grew. The collisions ceased only when the control distance *D*, for *n* = 20, 30 and 40, were equal to or higher than 2300, 4100 and 4700*m* respectively.

## Conclusion and proposals

The computational model presented replicates perfectly the conditions needed to efficiently manage AVs on simple road intersections and serves as a base for new models for more complex intersections. The simulations had satisfactory results in most cases, that is, there were no collisions when managing 20, 30, and 40 automated vehicles in conflicting directions crossing an intersection. However, the developed algorithm focused on finding a solution for the presented model that avoids collisions in the intersection, without considering the search for a solution that maximizes the objective function of the model. This is because the time to find a solution is limited by the time it takes for the vehicle to begin crossing the road. In other words, the solution needed to be presented in real-time, but could not eliminate all possible collisions for all control distances *D* considered.

This research demonstrates empirically that a controller can propose time-space intervals for each vehicle crossing the intersection, ensuring safe crossing. Since the time-space interval is proposed by the controller, there is potential for the controller to minimize the travel time of vehicles in real-time. However, the main limitation in this case will be the computational capacity of the controller. Others research that has worked with AV management at intersections uses a different method to ensure a space-time interval for vehicles at intersections, as they work based on acceptance and rejection protocols. Thus, the AV requests a space-time reservation at the intersection and the controller responds whether to grant or reject the request, with the latter case leaving the vehicle with its request pending in a waiting queue.

By observing the method presented in Fig 5, it is possible to notice that it does not guarantee the end of all collisions in a single iteration. Thus, in the next iteration, the same pair of vehicles might appear again in the new list of possible collisions, which is the reason the number of collisions is equal to zero when *D* is equal or superior to 2300 *m*, since the higher the control distance, the more time the algorithm has to adjust the vehicles’ speeds. For this reason, the presented method is efficient only when applied on roads where there is a considerable distance before reaching an intersection. One proposal to eliminate collisions completely, even for the shortest control distance *D* considered, is to improve the decision-making process or reduce the time increment of 1 second to a fraction of this time during iterations. Thus, the discrete model can resemble a continuous model as the time increment tends to zero. As a result, vehicle velocities would stabilize more quickly, reducing the discomfort of car occupants [20]. However, the time increment should be consistent with the communication time between the controller and the vehicle, as well as the response time of the automobile. Therefore, as technology advances in this regard, the time increment may be reduced.

In light of the above, there is room for new research to propose more efficient optimization methods to solve the presented model, considering maximizing its objective function. The developed method’s strength lies in its ability to solve the model in real-time for different vehicle lengths without the need for vehicles to stop in the holding area. However, the method’s drawback is the requirement for a long control distance to ensure its reliability. Future works should consider including left and right turns in the model, as presented by [21] or [22], so that the maneuver resembles how a human driver performs it.
